# Ultrastructural and physiological responses of potato (*Solanum tuberosum* L.) plantlets to gradient saline stress

**DOI:** 10.3389/fpls.2014.00787

**Published:** 2015-01-13

**Authors:** Hui-Juan Gao, Hong-Yu Yang, Jiang-Ping Bai, Xin-Yue Liang, Yan Lou, Jun-Lian Zhang, Di Wang, Jin-Lin Zhang, Shu-Qi Niu, Ying-Long Chen

**Affiliations:** ^1^Gansu Key Laboratories of Crop Genetic and Germplasm Enhancement and Aridland Crop Science, College of Agronomy, Gansu Agricultural UniversityLanzhou, China; ^2^Department of Chemistry, School of Chemistry and Chemical Engineering, Nanjing UniversityNanjing, China; ^3^State Key Laboratory of Grassland Agro-ecosystems, College of Pastoral Agriculture Science and Technology, Lanzhou UniversityLanzhou, China; ^4^Plant Nutrition and Soil Science and UWA Institute of Agriculture, School of Earth and Environment, The University of Western AustraliaPerth, WA, Australia; ^5^State Key Laboratory of Soil Erosion and Dryland Farming on the Loess Plateau, Institute of Soil and Water Conservation, Chinese Academy of Sciences and Ministry of Education, Northwest A&F UniversityYangling, China

**Keywords:** potato plantlets, saline stress, ultrastructure, antioxidant defense system, ion distribution

## Abstract

Salinity is one of the major abiotic stresses that impacts plant growth and reduces the productivity of field crops. Compared to field plants, test tube plantlets offer a direct and fast approach to investigate the mechanism of salt tolerance. Here we examined the ultrastructural and physiological responses of potato (*Solanum tuberosum* L. c.v. “Longshu No. 3”) plantlets to gradient saline stress (0, 25, 50, 100, and 200 mM NaCl) with two consequent observations (2 and 6 weeks, respectively). The results showed that, with the increase of external NaCl concentration and the duration of treatments, (1) the number of chloroplasts and cell intercellular spaces markedly decreased, (2) cell walls were thickened and even ruptured, (3) mesophyll cells and chloroplasts were gradually damaged to a complete disorganization containing more starch, (4) leaf Na and Cl contents increased while leaf K content decreased, (5) leaf proline content and the activities of catalase (CAT) and superoxide dismutase (SOD) increased significantly, and (6) leaf malondialdehyde (MDA) content increased significantly and stomatal area and chlorophyll content decline were also detected. Severe salt stress (200 mM NaCl) inhibited plantlet growth. These results indicated that potato plantlets adapt to salt stress to some extent through accumulating osmoprotectants, such as proline, increasing the activities of antioxidant enzymes, such as CAT and SOD. The outcomes of this study provide ultrastructural and physiological insights into characterizing potential damages induced by salt stress for selecting salt-tolerant potato cultivars.

## Introduction

As a major abiotic stresses, salinity affects plant growth and significantly reduces crop yield (Zhang et al., 2010; Zhang and Shi, 2013; Deinlein et al., 2014; Shabala et al., 2014). High soil salinity can lead to osmotic imbalance, ion-specific toxicity, alteration of composition and structure of membranes, and disruption of photosynthesis (Hasegawa et al., 2000; Zhang and Shi, 2013; Maathuis et al., 2014; Cabot et al., 2014; Zhang et al., 2014). Plants generally develop salt resistance mechanism and unique structures to survive under high saline-stress conditions (Deinlein et al., 2014; Gupta and Huang, 2014; Roy et al., 2014; Shabala et al., 2014). Therefore, a better understanding of the structural variations, ion distribution and physiological changes in crop plants induced by salinity should facilitate the identification of saline tolerance mechanisms (Roy et al., 2014).

Potato (*Solanum tuberosum* L.), as the fourth most important food crop in the world, has been identified as moderately salt-sensitive or salt-tolerant (Katerji et al., 2000). Under 50 mM NaCl treatment, potato growth decreased and tuber yield reduced to about 50%, while the growth of plants is completely inhibited at 150 mM NaCl (Hmida-Sayari et al., 2005). Bruns and Hecht-Buchholz (1990) found that the salt-induced changes were mainly observed in the chloroplasts, especially in the thylakoids. Different potato cultivars reacted differently to salt stress. Mitsuya et al. (2000) found the degradation of thylakoid membranes of chloroplast of sweet potato *in vitro* resulting from salt-induced oxidative stress (0 and 80 mM). In addition, ultrastructural changes at the cellular level in a salt-adapted potato callus lines grown in 150 mM NaCl (Queirós et al., 2011) demonstrated that salt-adapted potato cell line contained more large starch, reduced membrane system and no vesicles. Although the ultrastructural alterations induced by saline have been reported in many plant cells (Yamane et al., 2004; Miyake et al., 2006; Ferreira and Lima-Costa, 2008; Bennici and Tani, 2009, 2012), information regarding the effects of salinity on potato cells cultured *in vitro* is not specified and is incomplete.

Plants could sense changes of external environment and adapt to new conditions (Vij and Tyagi, 2007; Cabot et al., 2014; Deinlein et al., 2014). Plants have developed complex physiological and biochemical mechanisms to maintain a stable intracellular environment through accumulating various antioxidant enzymes and solute under salt stress (Wang et al., 2007; Zhang and Shi, 2013; Gupta and Huang, 2014; Roy et al., 2014). The osmotic adjustment in plant can maintain water uptake and cell turgor, allowing regular physiological metabolism (Serraj and Sinclair, 2002; Han et al., 2014). Proline, as an important osmosis protective agent, contributes to osmotic adjustment, protecting cells from damage (Silva-Ortega et al., 2008; Abrahám et al., 2010; Hou et al., 2013; Bojorquez-quintal et al., 2014; Gupta and Huang, 2014). Salt stress also caused overproduction of reactive oxygen species (ROS), leading to secondary oxidative stress (Nounjan et al., 2012; Mishra et al., 2011). ROS mainly generated from chloroplasts and mitochondria (Munns and Tester, 2008), attributed to membrane damage (Abdullahil-Baque et al., 2010), decrease of protein synthesis and inactivation of enzymes, seriously disrupting cell normal metabolism and inducing lipid peroxidation (Csiszár et al., 2012). Malondialdehyde (MDA) as a product of membrane lipid peroxidation could reflects oxidative damage to cell membrane (Koca et al., 2006; Yazici et al., 2007; Han et al., 2014). To avoid ROS-induced oxidative damage, plants could form antioxidant defense system to remove free radical and effectively avoid oxidative damage. Therefore, the increase of catalase (CAT) and superoxide dismutase (SOD) activity is correlated to the tolerance of plant to abiotic stresses (Hernández et al., 1993; Hossain et al., 2004; Daneshmand et al., 2010). Salt-tolerant potato could evolve a better protective mechanisms to detoxifying ROS by increasing the activity of antioxidant enzymes and content of proline (Arbona et al., 2008; Cho et al., 2012).

Higher accumulation of salt ions in leaves is very harmful for plant growth (Neocleous and Vasilakakis, 2007; Sabra et al., 2012; Khayyat et al., 2014; Liu et al., 2014a). Naeini et al. (2006) reported that more Na^+^ accumulated in roots and more Cl^−^ in leaves of pomegranates (*Punica granatum*) exposed to salt stress. Soil salinity usually reduces K^+^ uptake by roots of higher plants (Zhang et al., 2010; Maathuis et al., 2014). Recent research suggests that maintaining a high level of K^+^/Na^+^ ratio is important to salt tolerance in glycophytes (Maathuis and Amtmann, 1999; Carden et al., 2003; Peng et al., 2004; Lv et al., 2011; Maathuis et al., 2014). A number of studies have demonstrated that salinity also reduced Ca^2+^ absorption and transportation in plant (Tattini and Traversi, 2009; Evelin et al., 2012; Zhang and Shi, 2013; Liu et al., 2014a). Ca^2+^ has vital signal transduction function triggered by various environmental stresses. Especially, Ca^2+^ could alleviate Na^+^ toxicity on plants and has a regulation effect on ion selectivity absorption and transport (Zhu, 2002; Ben-Amor et al., 2010). Ca^2+^ is an essential component of the middle lamella and cell walls which participates in maintaining the stability of cell membrane, cell wall, and membrane-bound proteins, preventing membrane damage and leakage, and stabilizing wall structure (Maathuis and Amtmann, 1999; Liu et al., 2014a). Scanning electron microscope (SEM) equipped with energy dispersive X-ray Spectroscopy (EDX) has been extensively utilized for analysis of the elements distributed in plant tissues. Moreover, ion concentrations analyzed by EDX is comparable to that derived from atomic absorption or flame photometry of whole samples (Ebrahimi and Bhatla, 2011, 2012).

The present study was to investigate the anatomical response, ion distribution and physiological changes of potato plants to gradient salt (NaCl). Test tube plantlets were used in this study to allow a direct and fast approach to examine the physiological and biochemical mechanisms of salt tolerance. The present study will provide the insight of the anatomical response, in addition to physiological response, of *in vitro* propagated potato plantlets exposed to saline stress, and develop a useful method for screening salt-tolerant cultivars.

## Materials and methods

### Plant material and treatments

A local potato cultivar “Longshu No. 3,” released in 2002 by Gansu Academy of Agricultural Sciences, China, was used in this study. This cultivar has been largely grown in Northwestern China because of its moderate resistance to low temperature, drought and salinity. Potato plantlets were propagated in solidified Murashige and Skoog (MS) medium. A total of 6 plantlets were cultured in each triangular flask under 16/8 h photoperiods at 200 μmol/m^2^/s and 23 ± 2°C. For salt stress treatment, plantlet stems with at least two leaves were transferred to the MS medium containing NaCl at concentrations of 0 (control), 25, 50, 100, and 200 mM, respectively. Root, stem and leaf samples were collected 2 or 6 weeks after treatments for analysis. There were six plantlets in six triangular flasks for each treatment.

### Transmission electron microscopy

At each sampling time, the fully expanded uppermost leaves of potato plantlets were collected and fixed for 3 h at room temperature with 2% glutaraldehyde in 100 mM sodium cacodylate buffer with a pH value of 7.4 (Sabatini et al., 1963). Samples were post-treated in 1% (w/v) OsO_4_, similarly buffered for 6 h at room temperature, dehydrated in a graded ethanol series and propylene oxide, and infiltrated and embedded in Spurr's epoxy resin (Spurr, 1969). Ultrasections were obtained using a LKBV ultramicrotome and stained with uranyl acetate and lead phosphate. Images were observed and generated using a transmission electron microscope (JEM-1230 JEOL, Japan). The size of the intercellular space and cell wall was measured manually on the printed micrographs.

### X-ray microanalysis of ions

Root, shoot and leaf samples of each treatment were washed with distilled water, respectively. The middle sections of plant tissues were dipped in 5% agar, inserted to a depth of 1.0 cm in a copper holder, and sliced freehand with a razor blade to obtain transverse sections, and immediately frozen in liquid nitrogen. The samples were freeze-dried in vacuum and stored in a desiccator, followed by carbon coated with a high vacuum sputter coater and sputter-coated with gold in an argon atmosphere. Samples were analyzed in an scanning electron microscope (JSM-5600LV, JEOL, Japan) equipped with energy dispersive X-ray spectroscopy (INCA X-Max 80, Oxford Instruments) detector. The accelerating voltage was 10 kV. The counting time for each analysis was 60 s and the data were expressed as counts per second (cps) of an element peak after subtraction of the background. Then, these spectra were transformed to normalized data. All the detectable elements were transformed into the relative element weight. Counts per second of K, Na, and Cl were discerned by weight percentage in tissues. Five location spots of the same tissue of each section were analyzed.

### Physiological assays

Free proline and malondialdehyde content from plantlet were extracted and quantified following the ninhydrin-based colorimetric assays (Delauney et al., 1992) and thiobarbituric acid (Hodges et al., 2014), respectively. Activities of SOD and CAT were determined according to the ultraviolet absorption method assays of Giannopotitis and Ries (1977) and Stewart and Bewley (1980). To measure the stomatal aperture, leaf samples (2 × 2 mm) were collected from plantlets treated with or without NaCl stress. The lower epidermis of leaves was collected by scotch tape and examined under a compound Digital Microscope (Motic) after stained with 0.1% I-KI. The morphological parameters of stomata [guard cell length—L (μM) and guard cell width—W (μM)] magnified 200 ×, were measured with Motic Images Advanced 3.2. Stomatal area (S) was calculated as the product of L and W. Leaf chlorophyll content was determined spectrophotometrically in 80% acetone as described by Arnon (1949).

### Data analysis

Parameter data were presented as means with standard deviations (*n* = 6). Data were subjected to One-Way ANOVA and Duncan's multiple range tests for each parameter at *P* < 0.05 using SPSS 13.0.

## Results

### Effects of saline stress on the ultrastructure of leaf mesophyll cells

**Figure 1:**
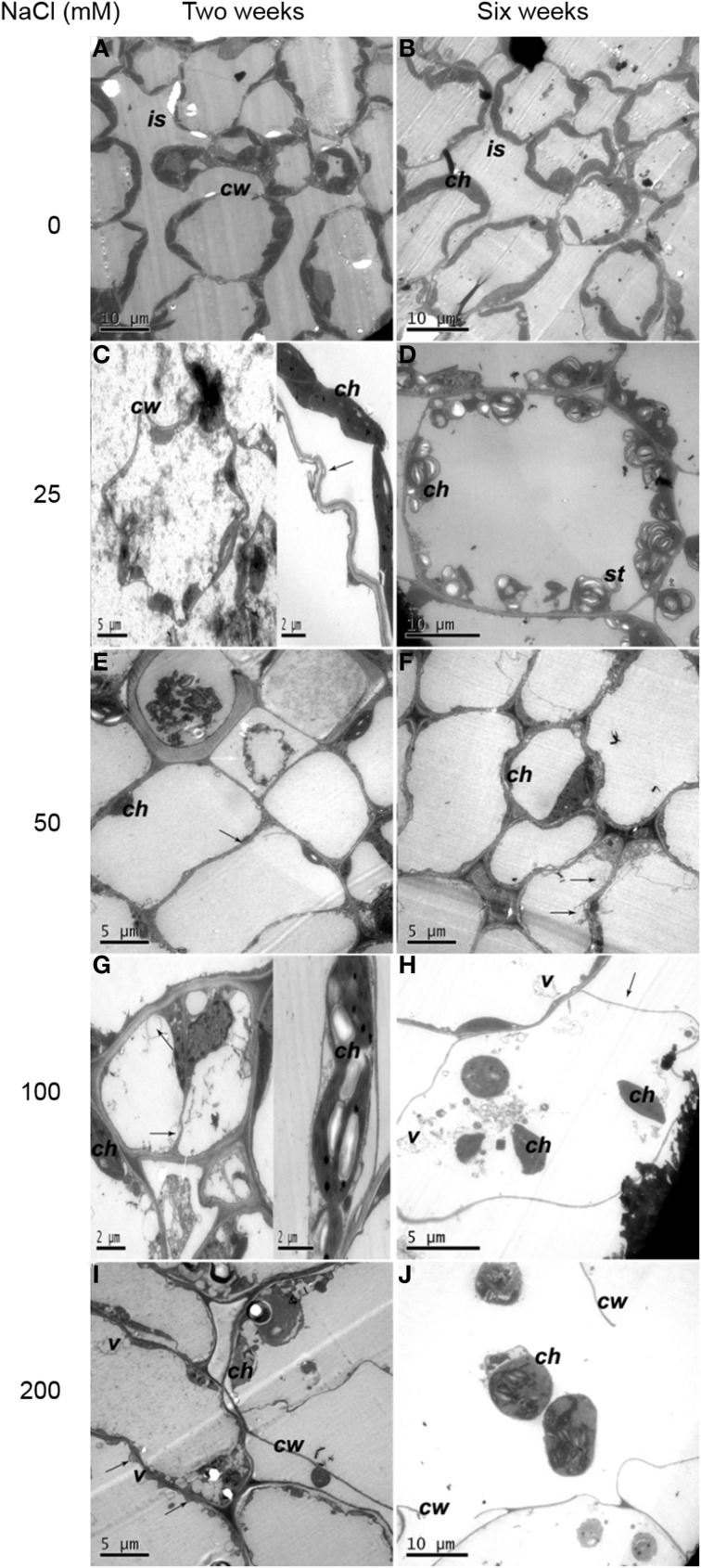
**Ultrastructural changes of mesophyll cells. (A)** Two weeks of non-salinity: intact mesophyll cells 2 weeks of non-salinity treatment. **(B)** Six weeks of non-salinity: more chloroplasts were present in mesophyll cells and cellular intercellular spaces increased for 6 weeks of 25 mM NaCl treatment. **(C)** Two weeks of 25 mM NaCl: cell walls were twisted, and the plasma membrane was apparently crimpled. Note chloroplasts were apart from the cell walls with membranous invaginations (black arrows). **(D)** Six weeks of 25 mM NaCl: mesophyll cell-contained chloroplasts have more starch grains. **(E)** Two weeks of 50 mM NaCl: mesophyll cells displayed plasmolysis (white arrow) and reduced intercellular spaces (black arrow). **(F)** Six weeks of 50 mM NaCl: complex vesiculation (black arrows), and dramatically reduced numbers of chloroplasts. **(G)** Two weeks of 100 mM NaCl: plasmolysis (white arrow), numerous vesicles (black arrows) and embedded chloroplasts. **(H)** Six weeks of 100 mM NaCl: cells showed severe plasmolysis (black arrows) and more vesicles and chloroplasts moved toward the cell center. **(I)** Two weeks of 200 mM NaCl: cells displayed severely damaged membrane systems, with severe membranous invagination (black arrow). **(J)** Six weeks of 200 mM NaCl: cell walls ruptured, and whole cells disintegrated. Note: ch, chloroplast; g, grana; pl, plastoglobuli; st, starch grains; w, cell wall; is, intercellular space; v, vesicle.

For 2 weeks of control plantlets (without salt stress), the ultrastructural distortion of mesophyll cells and chloroplasts was not observed. The structure of mesophyll cell was intact and the cell membrane was in close contact with the cell wall. Moreover, there was large intercellular space in mesophyll cells (Figure 1A). After 6 weeks growth, integrated chloroplasts of control plantlets were still closely arranged along plasma membrane (Figure 1B, Table 1).

**Table 1:** **Size of the Intercellular space and cell wall of the Mesophyll cell**.

**NaCl (mM)**	**0**	**25**	**50**	**100**	**200**
Intercellular space (μm)	6.41 ± 0.57 a	2.34 ± 0.07 b	0 ± 0 c	0 ± 0 c	NA
Cell wall (μm)	0.18 ± 0.02 a	0.19 ± 0.01 a	0.18 ± 0.00 a	0.26 ± 0.02 b	NA

*Values are means ± standard deviation (n = 6). Means in each line followed by different letters were statistically different (P < 0.05) by Duncan's multiple range tests. NA, not available. At 200 mM, parameters could not be obtained due to cell wall rupture and cell disintegration*.

For plantlets with 2 weeks of 25 mM NaCl treatment, mesophyll cell walls were twisted and plasma membrane crimpled remarkably. A small proportion of the chloroplasts with distended thylakoids were apart from the cell wall and membranous invagination was observed (Figure 1C). After 6-week treatment more starch grains were attached to the chloroplasts (Figure 1D) and intercellular space decreased (Table 1). For plantlets grown in 50 mM NaCl for 2 weeks, mesophyll cells showed some alterations (Figure 1E). The number of chloroplast decreased dramatically. Plasmolysis in some cells was accompanied by a reduction in mesophyll intercellular spaces. Six weeks later, chloroplasts showed irregular shape and complex vesiculation in the vacuoles was observed. Moreover, a number of cells appeared to be linked together without space (Figure 1F, Table 1). When plantlets were exposed to 100 mM NaCl for 2 weeks, serious plasmolysis was observed. Membranous invaginations resulted in numerous vesicles. Some chloroplasts embedded together (Figure 1G). Six weeks later, plasmolysis occurred severely accompanied by the presence of more vesicles in the vacuole. Chloroplasts moved toward the center of the cell (Figure 1H). The most dramatic alterations were observed in plantlets treated with 200 mM NaCl for 2 weeks. Membrane structure was severely damaged, characterized by severe membranous invagination (Figure 1I). After 6 weeks of 200 mM NaCl treatment, cell walls ruptured and the whole cell disorganized (Figure 1J).

### Effects of saline stress on the ultrastructure of chloroplasts

**Figure 2:**
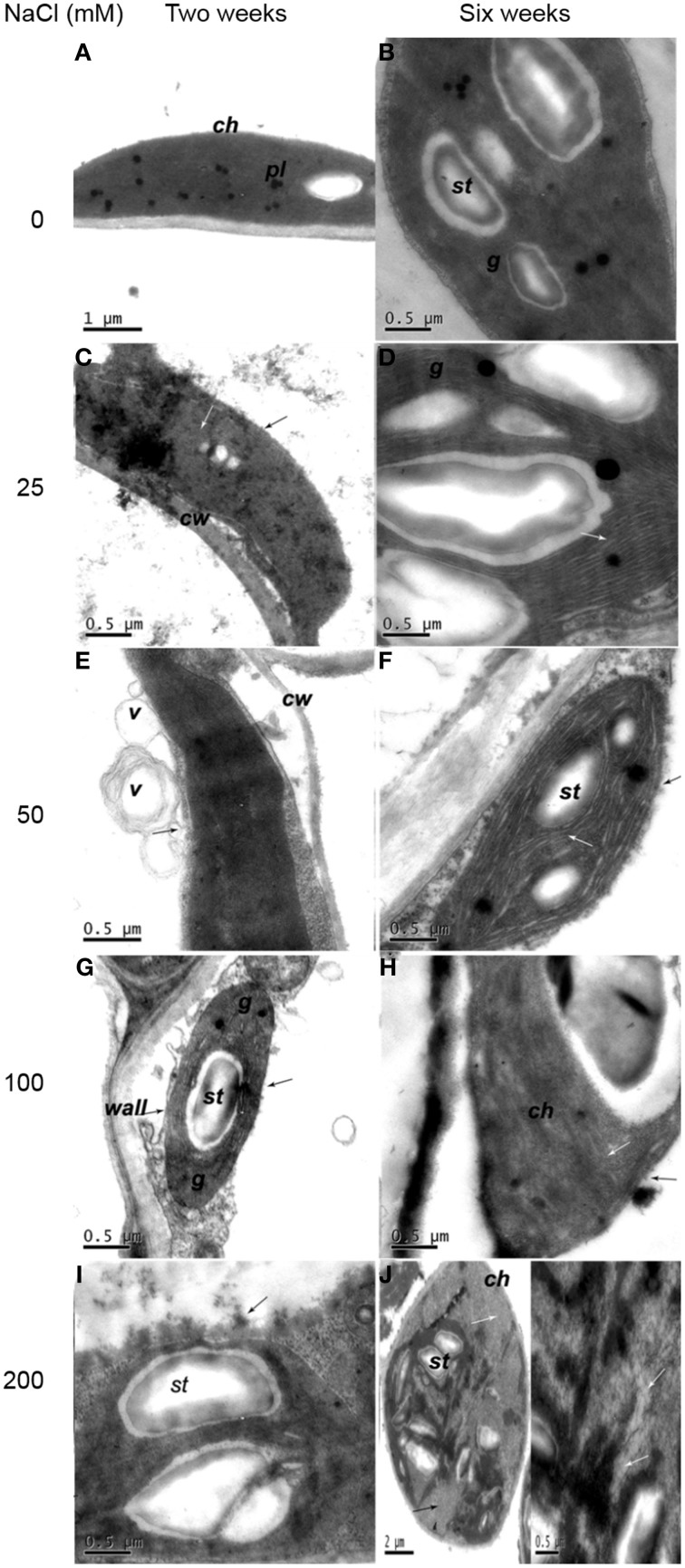
**Ultrastructural changes of chloroplast in mesophyll cell. (A)** Two weeks of non-salinity: ellipse- or spindle-shaped chloroplast with few and small starch. **(B)** Six weeks of non-salinity: chloroplast structure was complete. **(C)** Two weeks of 25 mM NaCl: chloroplast with vague outer membranes (black arrows) showed distended thylakoids (white arrows). **(D)** Six weeks of 25 mM NaCl: obvious swelling of the thylakoid (white arrow). **(E)** Two weeks of 50 mM NaCl: chloroplast envelope evagination, forming vesicles (black arrow). **(F)** Two weeks of 50 mM NaCl: chloroplast envelope disruption (black arrow) and distorted lamella (white arrow). **(G)** Two weeks of 100 mM NaCl: chloroplast envelope disintegration (black arrow) and thicker cell walls and partially dissolved grana thylakoid. **(H)** Six weeks of 100 mM NaCl: envelope (black arrow) and lamellar structure (white arrow) partly dissolved. **(I)** Two weeks of 200 mM NaCl: chloroplast disintegrated with inclusions effused (black arrows). **(J)** Six weeks of 200 mM NaCl: the grana and stromal lamella of chloroplast digest basically (black arrow), while thylakoids disintegrate and cavitate gradually (white arrows). Note: ch, chloroplast; g, grana; pl, plastoglobuli; st, starch grains; w, cell wall; is, intercellular space; v, vesicle.

For 2 weeks of control plantlets, integrated chloroplasts with few and small starch, containing compactly arranged thylakoids and well compartmentalized grana stacks with distinct grana lamellaes parallel to the chloroplasts' long axes, were observed (Figure 2A). Six weeks later, the membrane system was complete. The grana and stromal lamellae of chloroplast closely arranged and compacted thylakoids (Figure 2B).

When exposed to 25 mM NaCl for 2 weeks, the cell walls were thickened (Figure 2C, Table 1). The outer membrane of the chloroplast was vague. After 6 weeks of 25 mM NaCl treatment, the swelling of the thylakoids became obvious. The arrangement of lamella remained consistent, but showed a slight bend (Figure 2D). After 2 weeks of 50 mM NaCl treatment, chloroplast envelope was partially fragmented and evaginated to form complex vesicles (Figure 2E). Six weeks later, chloroplast envelopes disrupted with outer membranes disorganized. Grana lamella loosened with severely swollen thylakoids and space between lamella increased (Figure 2F). For plantlets treated with 100 mM NaCl for 2 weeks, the cell walls were much thicker (Table 1). Chloroplast envelope disintegrated and the grana thylakoid dissolved partially with reduced grana stacking, characterized by the presence of enlarged plastoglobuli and starch grains (Figure 2G). Six week later, the orientation of grana changed. Lamellar stacking decreased and dissolved dramatically. Membrane system was indistinct (Figure 2H). The most serious impact was observed when plantlets were treated with 200 mM NaCl. Some chloroplasts disintegrated with inclusions effused for plantlets treated with 200 mM NaCl for 2 weeks (Figure 2I). Six weeks later, the grana and stromal lamella of round chloroplasts with some starch grains digested basically, thylakoid membranes adhered to each other, while thylakoids disintegrated, cavitated, and even gradually disappeared (Figure 2J).

### Effects of saline stress on ion distribution in potato plantlet tissues

**Figure 3:**
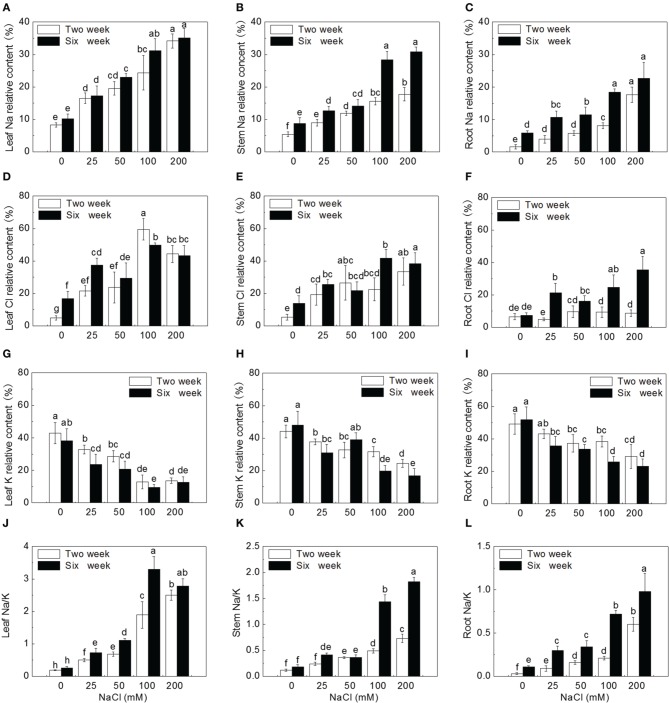
**Ion relative content and Na/K ratio under different concentrations of NaCl using SEM-EDS. (A)** Leaf Na relative content, **(B)** stem Na relative content, **(C)** root Na relative content, **(D)** leaf Cl relative content, **(E)** stem Cl relative content, **(F)** root Cl relative content, **(G)** leaf K relative content, **(H)** stem K relative content, **(I)** root K relative content, **(J)** ratio of Na to K in leaf, **(K)** ratio of Na to K in stem, **(L)** ratio of Na to K in root. Values are means and bars indicate SDs (*n* = 6). Columns with different letters indicate significant difference by Duncan's multiple range tests at *P* < 0.05 (Duncan test).

Na and Cl contents in leaves were relatively higher than that in stems and roots for all treatments. After 2 week treatments, Na relative content in leaves was 5.1, 4.2, 3.4, 3.0, and 1.9 times of that in roots at 0, 25, 50, 100, 200 mM NaCl treatments, respectively; Cl relative content in leaves was 1.2, 4.4, 2.5, 6.4, and 5.0 times of that in roots, respectively. After 6 week treatments, with the increase of NaCl in growth environment, the relative contents of Na and Cl in tissues were higher than those at 2 weeks, respectively. In addition, Cl relative content remained higher than Na content for the same treatment and for the same organ tissue, which follows the similar trend as at 2 weeks. After 6 week treatments, Na relative content in leaves was 1.7, 1.6, 2.0, 1.7, and 1.5 times of that in roots at 0, 25, 50, 100, 200 mM NaCl treatments, respectively; Cl relative content in leaves were 2.3, 1.7, 1.8, 2.0, and 1.2 times of that in roots at corresponding NaCl treatments, respectively. These results indicated that Na and Cl were mainly distributed in leaves of potato plantlets. (Figures 3A–F).

In contrast, K relative content in roots, stems and leaves showed a decreasing trend with the increase of external NaCl concentration. Accumulation of K in stems was reduced, particularly in leaves. After 2 weeks of salt treatment, K relative content in roots was 1.1, 1.3, 1.3, 3.0, and 2.1 times of that in leaves at 0, 25, 50, 100, 200 mM NaCl treatments, respectively. Six weeks later, K relative content in roots, stems and leaves decreased compared to that at 2 weeks. K relative content in roots was 1.3, 1.5, 1.6, 2.7, and 1.8 times of that in leaves at 0, 25, 50, 100, 200 mM NaCl treatments, respectively (Figures 3G–I). The comparison of K distribution in the different parts of potato plantlets showed that salinity seriously reduced K allocation toward leaves.

The Na/K ratio dramatically increased, especially in leaves after treated with various concentrations of NaCl. After 2 weeks of treatments, Na/K ratio significantly increased by 2.0, 4.3, 6.0, and 19.0 times in roots, 1, 2, 3.1, and 5.1 times in stems, and 1.6, 2.6, 8.9, and 12.1 times in leaves, at 25, 50, 100, 200 mM NaCl treatments, respectively, compared to that in control tissues, After 6-week treatment, compared to the corresponding organs of control plantlets, Na/K ratio significantly increased by 1.7, 2.1, 5.5, and 7.9 times in roots, 1.3, 1, 7, and 9.1 times in stems, and 1.8, 3.3, 11.7, and 9.7 times in leaves at corresponding NaCl treatments, respectively. Potato plantlets treated with salt for 6 weeks had higher Na/K ratio in the relevant organs than those treated for 2 weeks except for leaf Na/K ratio at 200 mM NaCl concentration (Figures 3J–L).

### Effects of saline stress on leaf free proline content, cat and SOD activities and MDA content

**Figure 4:**
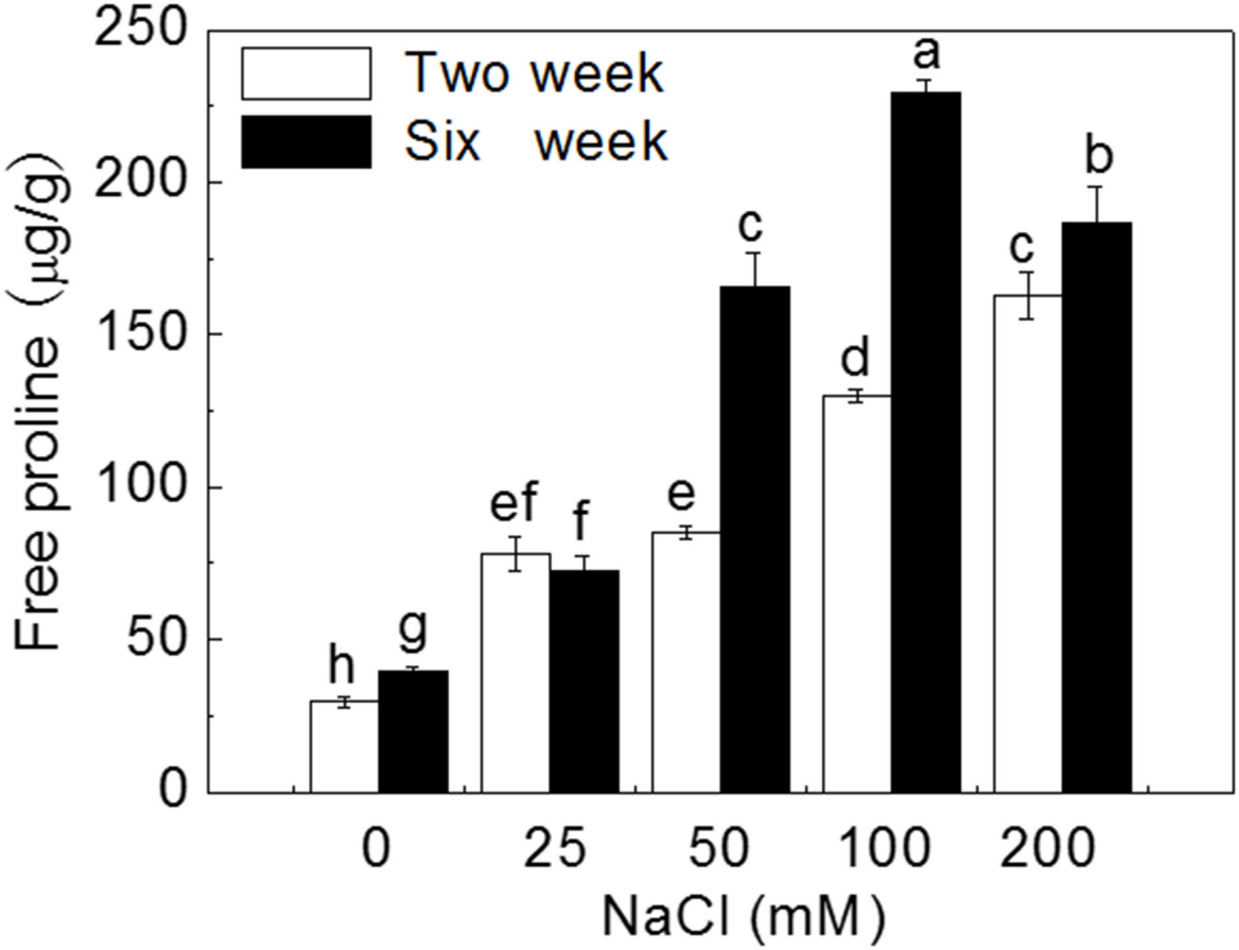
**Effects of NaCl treatment on free proline content**. Values are means and bars indicate SDs (*n* = 6). Columns with different letters indicate significant difference by Duncan's multiple range tests at *P* < 0.05.

Salt stress significantly increased free proline levels in leaves (Figure 4). After 2 weeks of treatment, proline content significantly increased by 1.6, 1.9, 3.4, and 4.5 times at 25, 50, 100, and 200 mM NaCl treatments, respectively, compared to control (*P* < 0.05). After 6 weeks of treatments, proline significantly content increased by 0.8, 3.1, 4.7, and 3.7 times, respectively (*P* < 0.05). Proline content decreased significantly at 200 mM NaCl compared to that at 100 Mm NaCl (*P* < 0.05). Leaf proline content in plantlets treated for 6 weeks by 50, 100, and 200 mM NaCl was significant higher than that in plantlets treated for 2 weeks (*P* < 0.05).

**Figure 5:**
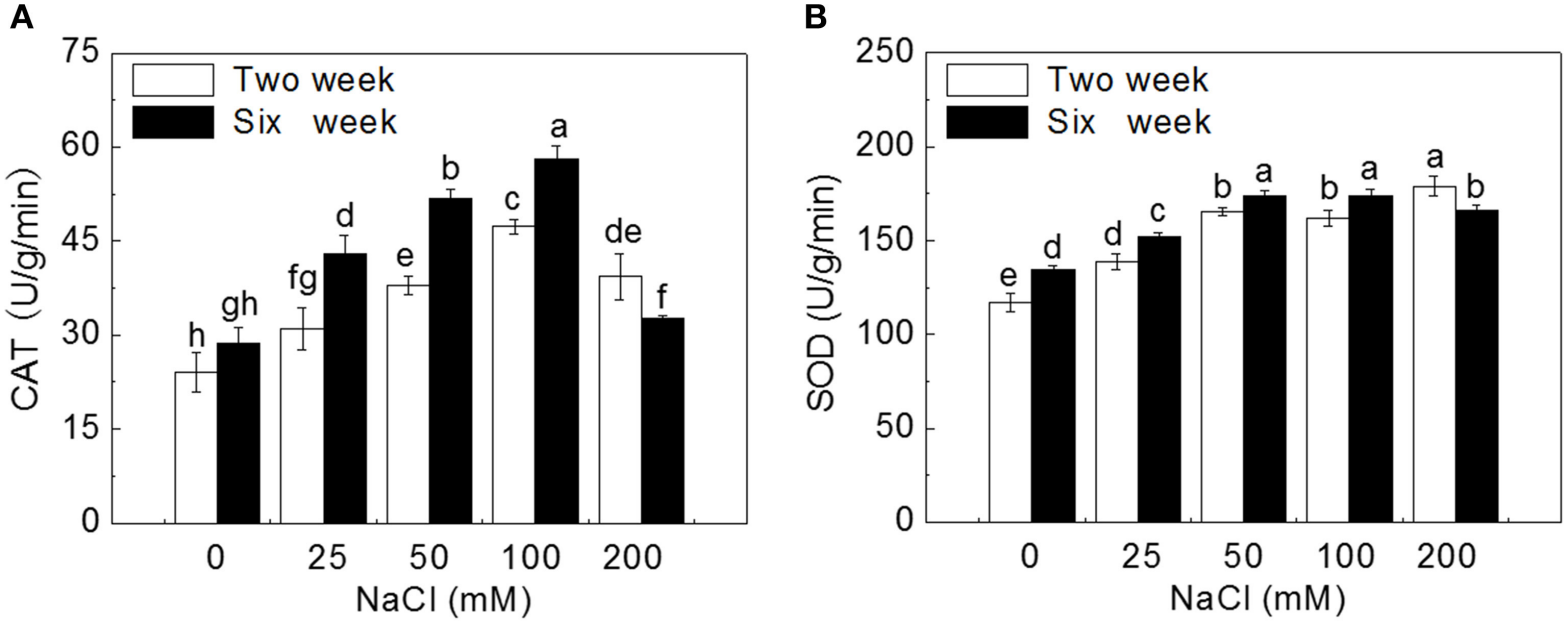
**Effects of NaCl treatment on activities of catalase (CAT) and superoxide dismutase (SOD)**. **(A)** CAT activity, **(B)** SOD activity. Values are means and bars indicate SDs (*n* = 6). Columns with different letters indicate significant difference by Duncan's multiple range tests at *P* < 0.05.

Salt stress increased the activity of the antioxidant enzymes. After 2 week treatment, compared to control, CAT activity significantly increased by 28.9, 57.9, 96.8, and 63.4% at 25, 50, 100, and 200 mM NaCl, respectively; while SOD activity significantly increased by 18.6, 41.2, 38.4, and 52.9%, respectively (*P* < 0.05). After 6 weeks, CAT and SOD activities significantly increased by 50.0, 80.5, 102.6, and 13.6%, and 13.1, 29.5, 29.6, and 23.9% at 25, 50, 100, and 200 mM NaCl, respectively, compared to corresponding control (*P* < 0.05). Leaf CAT activity in plantlets treated with 200 mM NaCl for 2 and 6 weeks and SOD activity for 6 weeks decreased significantly compared to that in plantlets treated with 100 mM NaCl (*P* < 0.05). Also, activities of leaf CAT and SOD in plantlets treated for 6 weeks were significantly higher than those in plantlets treated for 2 weeks except for 200 mM NaCl treatment (*P* < 0.05) (Figure 5).

**Figure 6:**
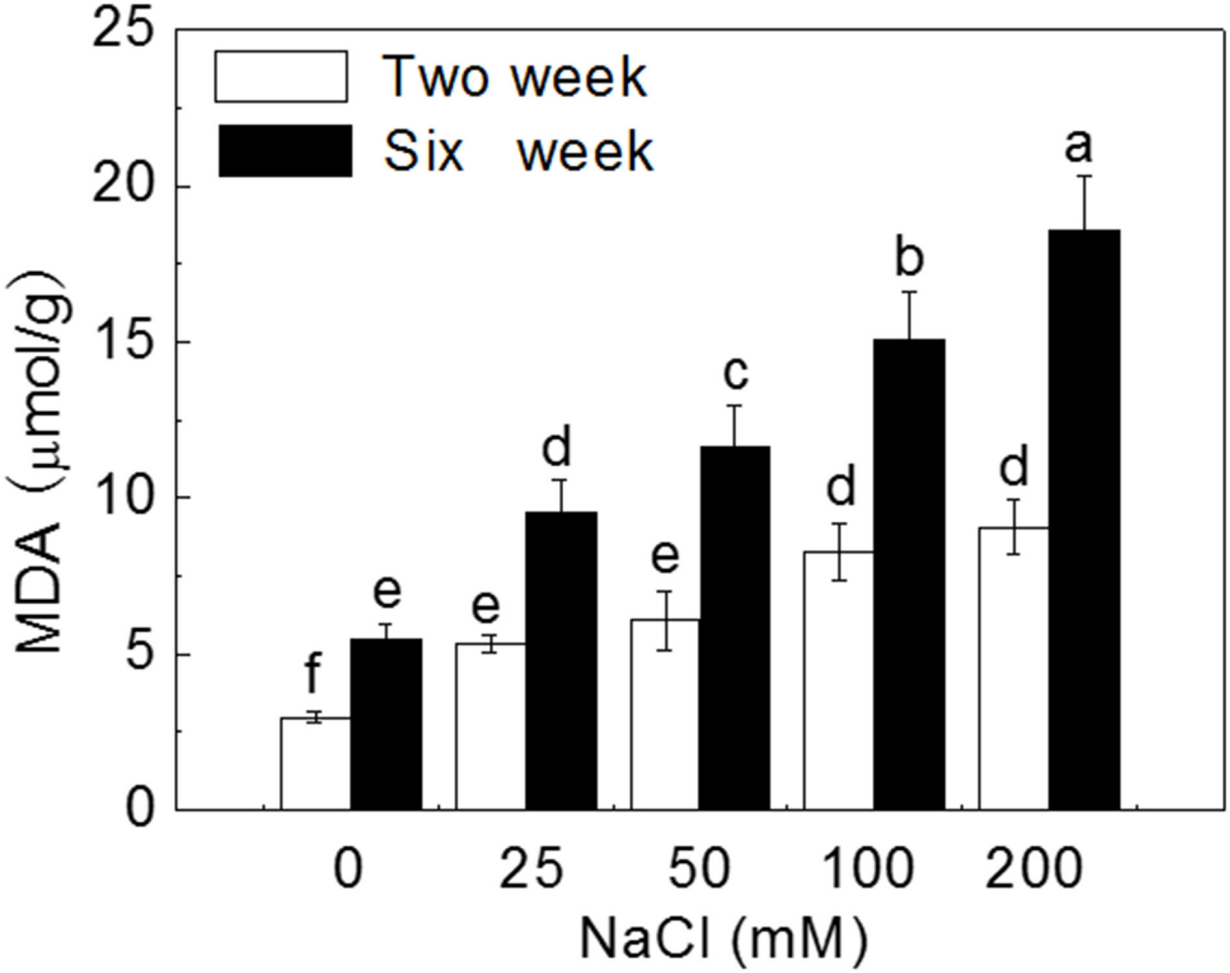
**Effects of NaCl treatment on malondialdehyde (MDA) content**. Values are means and bars indicate SDs (*n* = 6). Columns with different letters indicate significant difference by Duncan's multiple range tests at *P* < 0.05.

Leaf MDA content was used as an indicator of oxidative damage by salt stresses. After 2 week treatment, MDA content significantly increased by 0.8, 1.0, 1.8, and 2.0 times with the increase of external NaCl concentration compared to control plantlets; after 6 week treatment, MDA content sharply increased by 0.7, 1.1, 1.7, and 2.4 times with the increase of salinity (*P* < 0.05). Leaf MDA content in plantlets treated for 6 weeks were significantly higher than that in plantlets treated for 2 weeks (*P* < 0.05) (Figure 6).

### Effects of salinity stress on leaf stomatal area and chlorophyll content

**Figure 7:**
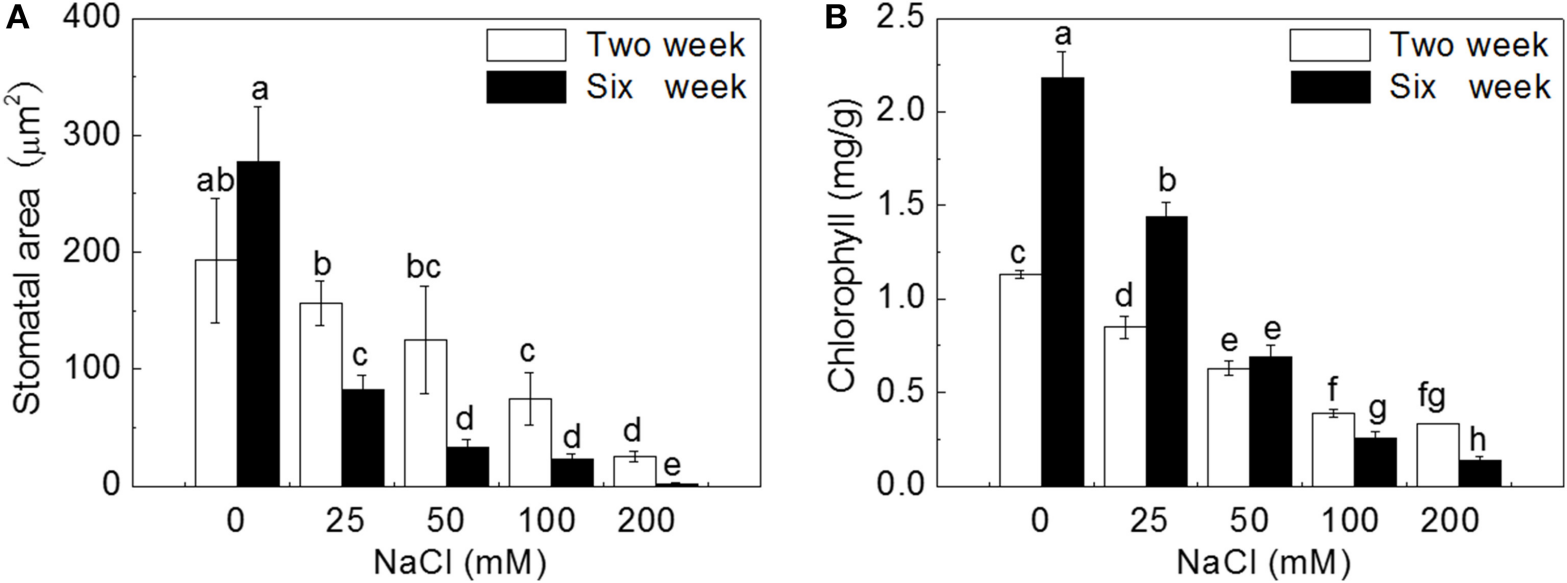
**Effects of NaCl treatment on stomatal area (A) and chlorophyll content (B)**. Values are means and bars indicate SDs (*n* = 6). Columns with different letters indicate significant difference by Duncan's multiple range tests at *P* < 0.05.

Two weeks of salt treatment reduced stomatal area significantly by 18.0, 35.4, 61.5, and 86.7% at 25, 50, 100, and 200 mM NaCl concentrations, respectively, compared to control (*P* < 0.05). Six weeks of salt treatment dramatically reduced stomatal area by 70.3, 88.2, 91.6, and 99.4% with the increase of NaCl concentration (*P* < 0.05). Stoma was almost closed after 6 weeks of 200 mM NaCl treatment (Figure 7A).

The trend of changes for chlorophyll content was similar to that for stomatal area. After 2 weeks of salt treatment, leaf chlorophyll content decreased gradually by 24.8, 44.2, 65.5, and 70.8% with the increase of NaCl concentration, compared to control (*P* < 0.05). After 6 weeks of salt treatment, chlorophyll content sharply decreased by 33.9, 68.3, 88.1, and 93.6% with the increase of NaCl concentration (*P* < 0.05), and was much lower than that at 2 weeks under corresponding salt stresses (Figure 7B).

At the whole plantlet level, NaCl treatments inhibited potato plantlet growth. The height of seedlings gradually decreased with increase of external NaCl concentration. After 6 weeks of treatment, severe salt stress (200 mM NaCl) induced a greater decline in shoot growth and root development of potato plantlets (Figure S1).

## Discussion

### Salinity induced ultrastructural changes of leaf mesophyll cells and chloroplasts

In present study, high levels of Na and Cl, and low level of K were distributed in leaves. The changes in chemical contents could result in ultrastructural alteration in leaf cells. Three salt-stress related alterations were observed. Firstly, the number of chloroplasts displaying swelled and distorted thylakoids decreased, accompanied by chloroplasts moving to the cell center. This chloroplast change is a typical effect of salinity as previously observed in salt-stressed *Cucumis sativus* L. (Shu et al., 2013). Secondly, cell walls thickened and plasmolysis occurred and the intercellular spaces of cell decreased with the increase of external salt concentration, which was also reported in potato cultivars (Bruns and Hecht-Buchholz, 1990; Navarro et al., 2007). Thirdly, lamella became disordered, loosened, and even indistinct, with reduced grana stacking because of inhibition of protein synthesis. Krzesłowska (2010) has reported that thickened cell wall could be as a barrier, protecting cell from toxicity of trace metals. So cell wall may function and limit passive Na and Cl enter into protoplast, maintaining structural integrity of the cell in the early low salt stress. It has been known salt stress can lead to osmotic damage. Na^+^ could be used directly for osmotic adjustment to maintain cell turgor and photosynthetic activity under low external salt concentration (Yousfi et al., 2010; Ebrahimi and Bhatla, 2012; Ma et al., 2012). However, with the increase of salt levels (NaCl concentration >50 mM), high concentrations of Na and Cl accumulated in leaf apoplast, leading to water loss of cell, plasmolysis and decrease of intercellular spaces in the leaves of potato plantlets. The present study observed invaginated membrane system forming numerous vesicles under salt treatments supporting observations by Kim and Park (2010), whilst contrary to Queirós et al. (2011) in which no vesicle was found in salt-adapted potato cell line. Vacuolation may be a response to membrane system damage induced by ROS caused by toxicity of Na and Cl (Kim and Park, 2010). ROS lead to the increase of plasma membrane permeability and extravasations of soluble substances, causing osmotic water imbalance, aggravating plasmolysis. Since membrane vesicles have Na^+^/H^+^ antiporter (Blumwald et al., 2000) and cell can sequester ion into vacuole (Kim and Park, 2010), vesicles may compartmentalize Na and Cl and migrate to walls. When plants were exposed to high NaCl concentration (100 mM), membrane disappeared. Salt inhibits absorption of Ca^2+^, further leading to instability of cell membrane and cell wall. Integral of membrane is essential in ions absorption and distribution. The destruction of the membrane structure inevitably disrupted ion homeostasis, affecting osmotic potential and inducing ion toxicity.

Disorganization of whole cells was accompanied by disintegrated chloroplasts having more starch and dissolved stroma lamella under 200 mM NaCl. It was speculated that starch synthesis plays a role in lessening the hyperosmotic stress as osmoticum. A total disorganization of the protoplast in callus cells was reported in other plants, possibly caused by dehydration (Bennici and Tani, 2012). Disintegration of chloroplasts and mesophyll cells end the photosynthesis, thus, maintaining structural integrity is necessary in plant growth (Bennici and Tani, 2012).

### Salinity changed ion homeostasis in potato plantlets

It has been known that the total Na^+^ and Cl^−^ content increased under salt in potato cell line, and K^+^/ Na^+^ ratio was a little higher in the adapted line (Queirós et al., 2011). Ruan et al. (2005) showed that Na^+^ accumulation decreased from the roots to leaves in *Kosteletzkya virginica*. Higher Na^+^ distributed in roots than in leaves in maize under salt stress (Azevedo-Neto and Prisco, 2004). In *Capsicum chinense*, more Na^+^ was restricted in roots (Bojorquez-quintal et al., 2014). Higher levels of Na^+^ in roots can maintain the normal osmotic potential and prevent it from being transported to the leaves, therefore avoiding the accumulation of Na^+^ in the leaves (Tester and Davenport, 2003; Munns and Tester, 2008; Xue et al., 2013). Queirós et al. (2009) reported that higher Na^+^ distributed in roots, inhibiting Na^+^ transport to leaves in potato cell. In present study, the distribution of Na and Cl increased from roots to stems and leaves in potato plantlets, indicating that potato is not a salt exclusion plant and has lower capacity to retain saline ions in their roots. High ions in leaves leaded to osmotic damage and oxidative stress, affecting physiological and biochemical metabolism. In addition, as a whole more Cl accumulated in potato tissue than Na, indicating the absorption of Cl^−^ was higher than Na, which is similar to the findings in sunflower (Ebrahimi and Bhatla, 2011) and in Clions (Greenway and Munns, 1980). Higher Cl^−^ accumulation lead to more serious and instant damage under salt stress (Yao and Fang, 2008). In our study, the absorption of Na and Cl in roots, stems and leaves of potato plantlet was enhanced with the increases of NaCl concentration, and the relative contents of Na and Cl were the highest in leaves, and lowest in roots.

K^+^ participates in many cellular functions, such as protein synthesis, enzyme activation and osmotic regulation (Peng et al., 2004; Takahashi et al., 2007; Amtmann et al., 2008). Therefore, the regulation of K^+^ homeostasis plays a critical role in plant tolerance to abiotic stresses (Ashley et al., 2006; Wang and Wu, 2010; Demidchik, 2014; Anschütz et al., 2014; Shabala and Pottosin, 2014). Salinity induced plant nutritional disorders, such as the suppression of K^+^ uptake (Kader and Lindberg, 2005; Kronzucker et al., 2006; Shabala and Cuin, 2008). Bojorquez-quintal et al. (2014) suggested that more K^+^ accumulated in roots is correlated with the salt tolerance of *Capsicum chinense*. In present study, salt stress dramatically reduced K^+^ uptake and accumulation, especially in leaves, resulting in increased Na/K ratio in all tissues with the increase of external salt concentration and the duration of treatments.

### Physiological mechanism of potato plantlets adapting to gradient saline stress

Salinity leads to physiological changes in plant, especially osmotic and oxidative stress (Zhang and Shi, 2013). The accumulation of osmoprotectants is important for plant to adapt to osmotic stress (Apse and Blumwald, 2002; Waditee et al., 2007; Chan et al., 2011; Rivero et al., 2014). Proline, an important compatible osmolyte in plants, could maintain cell turgor and function in osmotic adjustment to improve plant tolerance to osmotic stress (Abrahám et al., 2010; Huang et al., 2013). In many plants, the accumulation of proline could lead to salt tolerance and has even been used as an important trait in selecting tolerant species or genotypes (Ashraf and Harris, 2004; Khelil et al., 2007; Ruffino et al., 2010). Recently, Bojorquez-quintal et al. (2014) found that more proline was accumulated in leaves of salt-tolerant habanero pepper (*Capsicum chinense* Jacq.) cultivar (Rex) than in salt-sensitive one (Chichen-Itza) under 150 mM NaCl treatment. In our study, the levels of free proline increased significantly with the increase of external salt concentration and with the duration of treatments except for a little decline at 200 mM NaCl after 6-week treatment (Figure 3). The reason may be that 200 mM induced excessive damage to plant cells and inhibited proline synthesis.

Antioxidant enzymes in plant can remove ROS and alleviate oxidative damage (Krantev et al., 2008; Mishra et al., 2011). It has been known that the higher activities of CAT and SOD could improve plant tolerance to salinity and K^+^-deficiency conditions (Wang et al., 2010; Zhou et al., 2014). It was found that SOD activity was significantly higher in the leaves of salt-tolerant wild tomato (*Lycopersicon pennellii*) than that of salt-sensitive cultivated tomato (*Lycopersicon esculentum*) after 12 and 84 d of salt treatment (140 mM NaCl) (Koca et al., 2006). Similarly, salt-tolerant *Plantago maritima* showed a better protection mechanism against oxidative damage caused by salt stress by its higher induced activities of CAT, SOD, glutathione reductase (GR) and peroxidase (POX) than the salt-sensitive *P. media* (Sekmen et al., 2007). Co-expression of the *Suaeda salsa* CAT and glutathione S-transferase (GST) genes enhanced the active oxygen-scavenging system that led to improved salt tolernace in transgenic rice, resulting from not only increased CAT and GST activities but also the combined increase in SOD activity (Zhao and Zhang, 2006). Jing et al. (2014) reported that overexpression of mangrove (*Kandelia candel*) copper/zinc superoxide dismutase gene (*KcCSD*) enhanced salinity tolerance in tobacco: *KcCSD*-transgenic lines were more Na^+^ tolerant than wild-type (WT) tobacco in terms of lipid peroxidation, root growth, and survival rate; Na^+^ injury to chloroplast was less pronounced in transgenic tobacco plants due to enhanced SOD activity by an increment in SOD isoenzymes under 100 mM NaCl stress from 24 h to 7 d; catalase activity rose in *KcCSD* overexpressing tobacco plants and transgenic plants better scavenged NaCl-elicited ROS compared to WT ones. In present study, the activities of CAT and SOD in leaves of potato plantlets significantly increased with the increase of NaCl concentration (0~100 mM) in medium. When exposed to 200 mM NaCl, especially after 6 weeks, leaf cells were severely damaged, even disorganized (Figure 1), leading to the damage of cellular structure or alterations of metabolism, and reducing the synthesis of CAT and SOD.

Soil salinity is known to increase the level of ROS in plant leaves and MDA is a major product of membrane lipid peroxidation (Mittova et al., 2004; Koca et al., 2006; Yazici et al., 2007). Therefore, leaf MDA content could represent the degree of cell membrane damage and is usually used to evaluate plant salt tolerance (Luna et al., 2000; Miao et al., 2010; Han et al., 2014). In our study, leaf MDA content increased significantly with the increase of external salt concentration after 2-week treatment and even increased more rapidly after 6-week treatment. However, the activities of SOD and CAT may not enough to eliminate ROS, resulted in large production of MDA under higher salt stress (200 mM).

### Salinity reduced leaf stomatal area and chlorophyll content

Chlorophyll is essential for photosynthesis, and the increase of chlorophyll content can reflect the increase of photosynthetic activity (Yamori et al., 2006). Ben et al. (2010) and Su et al. (2011) suggested that the accumulation of chlorophyll content could enhance plant salt tolerance. In the present study, leaf chlorophyll content gradually decreased with the increase of NaCl treatment and duration, which could result from the inhibition of chlorophyll synthesis caused by chloroplast damage.

Gas exchange through stoma play important role in carbon assimilation (Wilkinson and Davies, 2002). Salt stress decreases leaf stomatal area by reducing leaf water content and leaf turgor induced by ABA signal (Wilkinson and Davies, 2002). Therefore, stomatal conductance was correlated to salinity stress (Liu et al., 2014b). In our study, salt stress seriously induced stomatal closure. Reduced CO_2_ diffusion caused by stomatal closure lead to suppression of photosynthesis, affecting plant growth (Figure S1).

**Figure 8:**
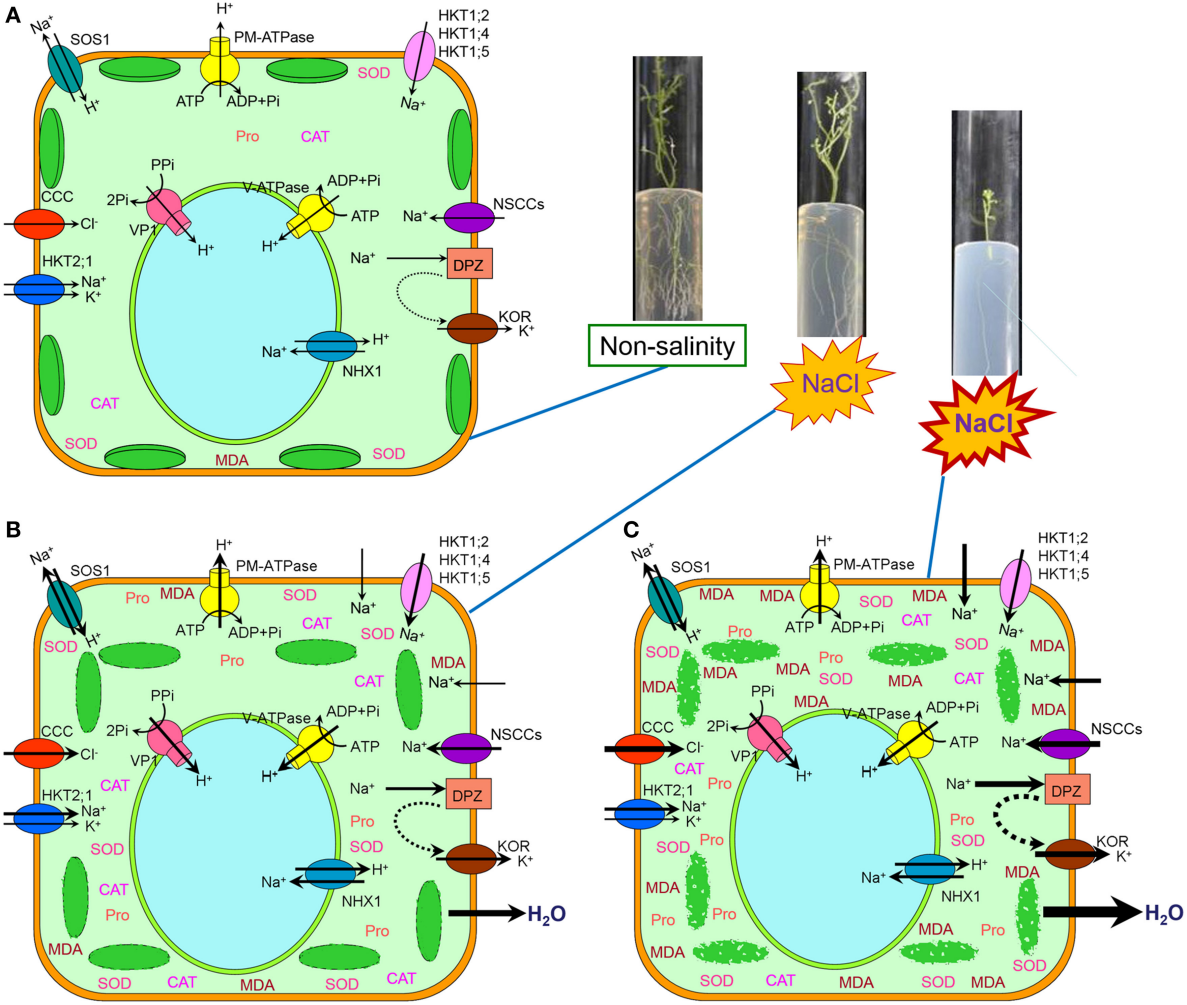
**Schematic model of ultrastructural and physiological responding mechanisms of the mesophyll cells from potato (*Solanum tuberosum* L.) plantlets to gradient saline stress. (A)** Under non-salinity condition, water and ions was maintained at a balance status, only little proline (Pro), CAT, SOD, and MDA were accumulated within cytosol, and integrated chloroplasts were closely arranged along plasma membrane. **(B)** Under moderate salinity condition, abundant Na^+^ was transported into cytosol probably through non-selective cation channels (NSCCs), high-affinity K^+^ transporters (HKTs, probably HKT1;2, HKT1;4, HKT1;5, and HKT2;1) and a little permeated directly across plasma membrane, and Cl^−^ was probably transported by cation-Cl^−^ cotransporter (CCC). Some K^+^ was transported out of the cell by K^+^ outward-rectifying channels (KORs) activated by membrane depolarization (DPZ). The membrane system was damaged resulting in the increase of MDA and damaged chloroplasts were not closely arranged along plasma membrane. Stoma closed because of water loss and chlorophyll content decreased because of chloroplast damage. For adaptation to moderate salinity, Na^+^ efflux or extrusion by plasma membrane Na^+^/H^+^ antiporter (salt overly sensitive, SOS1) motivated by plasma membrane ATPase (PM-ATPase) and vacuolar Na^+^ compartmentation by tonoplast Na^+^/H^+^ antiporter (NHX1) motivated by vacuolar ATPase (V-ATPase) and H^+^-pyrophosphatase (VP1) functioned to reduce Na^+^ toxicity in cytosol, at the same time osmoprotectants such as proline were accumulated and the activities of antioxidant enzymes (CAT and SOD) increased. **(C)** Under high salinity condition, more and more Na^+^ was transported into cytosol probably through NSCCs and permeated directly across plasma membrane although the amount of Na^+^ transported by HKTs did not increase, and more Cl^−^ was probably transported by CCC. More and more K^+^ was transported out of the cell by KOR. The membrane system was seriously damaged resulting in the rapid increase of MDA and disintegrated chloroplasts appeared. Stoma closed completely because of increasing water loss and chlorophyll content decreased dramatically because of severe chloroplast damage. However, the ability of Na^+^ efflux or extrusion by SOS1 and vacuolar Na^+^ compartmentation by NHX1 were not enhanced because of serious damage to membrane system, at the same time osmoprotectant content and the activities of antioxidant enzymes (CAT and SOD) did not increased any more, but even decreased. Therefore, the growth of potato plantlets was inhibited.

In conclusion, the adaptation of plants to salt stress is a complex process at cellular, biochemical and physiological levels. In the present study, several parameters were analyzed to demonstrate ultrastructural and physiological responding mechanisms of potato (*Solanum tuberosum* L.) plantlets to gradient saline stress (Figure 8). We found that with the increase of external NaCl concentration and the duration of treatments, the number of chloroplasts and cell intercellular space markedly decreased, cell wall thickened and even ruptured, and mesophyll cells and chloroplasts were gradually damaged to a complete disorganization. Above ultrastructural changes may be induced by the increased concentrations of Na^+^ that was transported into cytosol probably through non-selective cation channels (NSCCs), high-affinity K^+^ transporters (HKTs, probably HKT1;2; HKT1;4; HKT1;5 and HKT2;1) and permeated directly across plasma membrane, and Cl^−^ that was probably transported by cation-Cl^−^ cotransporter (CCC) (Apse and Blumwald, 2007; Plett and Moller, 2010; Zhang et al., 2010; Zhang and Shi, 2013; Almeida et al., 2014a,b; Maathuis, 2014; Maathuis et al., 2014). More and more K^+^ was probably transported out of the cell by K^+^ outward-rectifying channels (KORs) activated by membrane depolarization (DPZ) (Chen et al., 2007; Sun et al., 2009; Lu et al., 2013; Demidchik, 2014; Demidchik et al., 2014; Lai et al., 2014). Leaf MDA content increased significantly due to all membrane lipid peroxidation induced by increasing and continuous salt stress, which also induced stomata closure and chlorophyll content decline. Potato plantlets showed adaptation ability to moderate salt stress through Na^+^ efflux or extrusion by plasma membrane Na^+^/H^+^ antiporter (salt overly sensitive, SOS1) motivated by plasma membrane ATPase (PM-ATPase), vacuolar Na^+^ compartmentation by tonoplast Na^+^/H^+^ antiporter (NHX1) driven by vacuolar ATPase (V-ATPase) and H^+^-pyrophosphatase (VP1), accumulating osmoprotectants such as proline, and improving the activities of antioxidant enzymes (CAT and SOD). This work provided both anatomical and physiological data for characterization of damages induced by salinity and the method could be used for selecting salt-tolerant potato cultivars.

### Conflict of interest statement

The authors declare that the research was conducted in the absence of any commercial or financial relationships that could be construed as a potential conflict of interest.

## Supplementary material

Figure S1 **Growth of potato plantlets in MS agar plates**. Plantlets grown on MS were transferred to new solid agar MS supplemented with various concentrations of NaCl (0, 25, 50, 100, and 200 mM) for 2 and 6 weeks, respectively.
