# Supplementary figures and images for: Ultrastructural and physiological responses of potato (Solanum tuberosum L.) plantlets to gradient saline stress
Source: Front Plant Sci. 2015 Jan 13;5:787. doi: 10.3389/fpls.2014.00787 (PMC4292236; doi:10.3389/fpls.2014.00787)

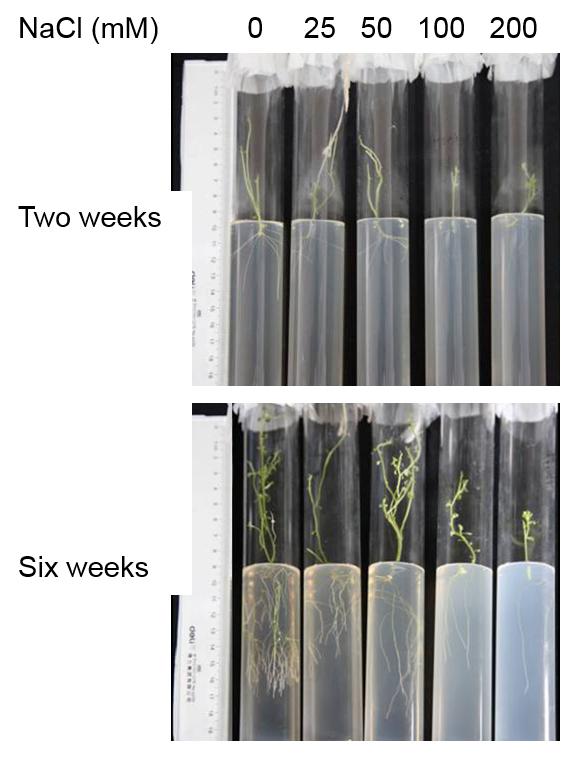

Supplement: Figure S1 — Growth of potato plantlets in MS agar plates. Plantlets grown on MS were transferred to new solid agar MS supplemented with various concentrations of NaCl (0, 25, 50, 100, and 200 mM) for 2 and 6 weeks, respectively. [file Image1.TIF]
